# Composite *Fasciola hepatica* faecal egg sedimentation test for cattle

**DOI:** 10.1136/vr.105128

**Published:** 2019-02-02

**Authors:** John Graham-Brown, Diana J L Williams, Philip Skuce, Ruth N Zadoks, Stuart Dawes, Harry Swales, Jan Van Dijk

**Affiliations:** 1 Infection Biology, University of Liverpool Institute of Infection and Global Health, Liverpool, UK; 2 Moredun Research Institute, Midlothian, UK; 3 University of Glasgow Institute of Biodiversity Animal Health and Comparative Medicine, Glasgow, UK; 4 Institute of Veterinary Science, Liverpool, Merseyside, UK; 5 Epidemiology and Surveillance, Centre for Preventive Medicine, Animal Health Trust, Newmarket, Suffolk, UK

**Keywords:** liver fluke, diagnostics, cattle, parasitology, herd health

## Abstract

Options for diagnosing *Fasciola hepatica* infection in groups of cattle are limited. Increasing the opportunities for herd-level diagnosis is important for disease monitoring, making informed treatment decisions and for flukicide efficacy testing. The sensitivity of a simple sedimentation method based on composite faecal samples for the detection of fluke eggs in cattle was assessed through a combination of experimental and statistical modelling techniques. Initially, a composite sample method previously developed for sheep was used to investigate the sensitivity of composite sample testing compared with individual counts on the same samples in cattle. Following this, an optimised, validated, qualitative (presence-absence) composite sample field test was developed for cattle. Results showed that fluke egg counts obtained from a composite sample are representative of those expected from individual counts. The optimal sampling strategy was determined to be 10 individual 10 g samples (100 g composite sample) from which a 10 g subsample is taken for sedimentation. This method yielded a diagnostic sensitivity of 0.69 (95 per cent CI 0.5 to 0.85). These results demonstrate the validity and usefulness of a composite faecal egg sedimentation method for use in the diagnosis and control of *F. hepatica* in groups of cattle, with the caveat that a negative test should be followed up with a second test due to limitations relating to test sensitivity.

## Introduction

The digenean trematode, *Fasciola hepatica*, is an important parasite of grazing animals in the UK. On cattle farms, fasciolosis represents a major source of economic loss through impaired productivity, reduced weigh gain, poor reproductive performance, reduced milk yields^1–4^ and, in heavy infections, mortality.^5^ Carcass quality is also affected, and this can lead to condemnation of (parts of) finished animals at slaughter. For example, 2010 abattoir data show liver condemnations in 22 per cent of all (>2.2 million) bovine carcasses processed in Great Britain, at an estimated cost of £3.2 million.^6^

Disease surveillance data have indicated an increasing prevalence of fasciolosis in endemic regions^7^ and an emergence of *F. hepatica* in eastern parts of the UK where it had not previously been considered of major importance.^8 9^ Climate-driven predictive models suggest further increases in parasite abundance and changes in both the seasonality and temporal range of *F. hepatica* in the near future.^10 11^ This is particularly worrying considering the concurrent emergence of triclabendazole (TCBZ)-resistant *F. hepatica*,^12–15^ with vaccination remaining years away from commercial reality.^16 17^

The first step towards control of a parasite remains accurate diagnosis. Diagnostic tests are also used to guide the timing of anthelmintic treatments.^18^ Three main methods are commercially available in the UK to diagnose fluke infections in cattle: faecal egg counts (FEC), a coproantigen-based ELISA (cELISA) and an *F. hepatica*-specific antibody serum/milk ELISA. Each of these tests has relative strengths and weaknesses affecting suitability for use under different circumstances and for different objectives.

Due to its simplicity, faecal egg sedimentation is the most commonly used test in low-tech laboratory setups. Several methods to detect fluke eggs in individual faecal samples have been described,^19–22^ with wide ranging test sensitivities quoted for diagnosis in cattle (43 per cent–91.9 per cent).^20 23^ A clear strength of faecal egg counts is the very high test specificity (approaching 100 per cent) provided treatment history is known. Liver fluke eggs are easily identifiable even under low power microscopy (10–40x magnification), being ovoid in shape, measuring 130–150×60–90 µm and possessing a single operculum. *F. hepatica* eggs may also be distinguished from paramphistome (rumen fluke) eggs, which are also extracted by sedimentation, with the former being golden brown in colour as opposed to the latter’s clear uncoloured appearance. While the diagnostic performance of faecal egg sedimentation in cattle has to date only been evaluated for individual samples, a composite sample method has been validated in sheep for both flock/group-level diagnosis and evaluation of treatment efficacy.^24^

The cELISA is a commercially available sandwich ELISA (Bio X diagnostics, Belgium) that detects *F. hepatica-*specific excretory-secretory (E/S) antigen in faeces. In lambs, the test has been shown to identify infection in individual animals from 5 weeks postinfection, with test sensitivity approaching 100 per cent from 10 weeks postinfection onwards. The test initially also looked promising for cattle with Mezo and others^25^ reporting a cELISA test sensitivity of 93 per cent compared with post mortem as the gold standard. However, Duscher and others^26^ subsequently reported a relative sensitivity of only 30 per cent for the cELISA when compared with serum antibody ELISA in naturally infected cattle and a relative sensitivity of 40 per cent compared with fluke egg counts. Investigation of the cELISA using pooled faecal samples using a modified cut-off value to evaluate treatment efficacy in cattle has shown promise under experimental and field conditions,^27 28^ although currently the sensitivity and specificity of such testing remains undefined, limiting the value of this method for herd-level diagnosis in cattle. Similarly, an improved, more sensitive, version of the cELISA is now commercially available,^29^ although again this requires further evaluation for cattle in a field setting.^30^

The *F. hepatica*-specific antibody ELISA is capable of detecting IgG antibody against *F. hepatica* E/S antigen in individual bovine serum and milk samples from two to four weeks postinfection, with an overall test sensitivity and specificity of 98 per cent and 96 per cent, respectively.^31 32^ The antibody ELISA also has a clear practical advantage over both FECs and the cELISA, since it has been validated for use in herd-level diagnosis of lactating dairy cattle through bulk milk tank (BMT) sample analysis with a sensitivity and specificity of 96 per cent and 80 per cent, respectively.^33^

Overall, both ELISAs have a distinct practical advantage over faecal egg counts in terms of high-throughput testing, sample storage and processing, while the simplicity of FEC sedimentation methods offers a valid diagnostic option in more low-tech field and laboratory settings such as those in first opinion practice, minimising costs and reducing time required to reach a diagnosis. A recent study directly comparing performance of all three tests in naturally infected UK cattle suggests the antibody ELISA to be the most sensitive, but least specific overall.^34^ Similarly, faecal egg counts and the cELISA methods appeared comparable in terms of sensitivity and specificity, although sensitivity of faecal egg counts varied with time due the seasonal nature of *F. hepatica* transmission in the UK and the inability of fluke egg counts to detect prepatent infection.

While the BMT antibody ELISA presents a valid option for herd/group-level diagnosis in lactating dairy cattle, opportunities for diagnostic screening in groups of beef and non-lactating dairy cattle are limited, relying on sampling and testing multiple individual animals, which is time consuming and/or expensive. Milk samples are not readily available for such animals and blood samples comparatively difficult to obtain. Hence, there is a clear need for a simple, robust, inexpensive test based on faecal samples for qualitative herd/group-level diagnosis of *F. hepatica* infection in cattle. Such a test may be useful for diagnosis, monitoring of infection and evaluating disease control programmes. To meet this need, the authors have here developed and validated a composite faecal egg count test for cattle. First, the authors assess whether a composite count is as sensitive as individual sedimentation tests using the same animals. Second, the authors sample all the animals in a herd and calculate the sensitivity of the test when different numbers of animals are included in the composite sample.

## Materials and methods

### Fluke egg sedimentation

Fluke egg counts were performed on individual and composite samples using a standard sedimentation technique.^19^ Ahead of processing, samples were transported and stored at 4°C in sealed plastic bags to prevent development of *F. hepatica* eggs.^35^ All samples were processed within two weeks of collection.

Briefly, faeces (specific quantities used for individual and composite samples are specified under study design) were homogenised in tap water and passed through stacked sieves (Endecotts, London England) of large (750–800 µm), medium (150–200 µm) and small (38–55 µm) mesh sizes, respectively, then washed with copious volumes of water to thoroughly fragment the sample. Once water exiting the small mesh sieve ran clear, the top two sieves were removed and their contents discarded. The retentate on the small mesh sieve was then transferred to a glass beaker and diluted to a volume of 500 ml with tap water. The suspension was left to stand for four minutes to allow sedimentation of *F. hepatica* eggs following which the supernatant was decanted and the sediment re-suspended in 500 ml of water. This process was repeated until no suspension remained after four minutes. The final supernatant was then decanted and the sediment transferred to a petri-dish for microscopy.

All samples were counted using a stereo dissecting microscope at 10–40x magnification. To aid counting, 1 per cent (w/v) methylene blue was added to the sample to stain background material. Samples containing greater quantities of sediment were divided between two and three petri-dishes to ensure accurate counting.

### Study design

To develop a composite faecal egg count for qualitative diagnosis of *F. hepatica* infection in groups of cattle, two important but separate characteristics of composite samples were assessed.

#### Comparison of composite and multiple individual sedimentation test performance

Faecal samples (n=138) were collected from 7 beef herds (A.1 to A.7) with a herd size ranging between 32 and 230 (median 50) animals in the region surrounding Bala, Gwynedd, north Wales in March 2012. Cattle sampled were a mixture of adult suckler cows and their calves, with 19 store cattle also present on one farm (A.1). Treatment regimens for *F. hepatica* varied widely between farms, from no treatments administered in the case of two farms (A.3 and A.5) through to three times a year for one farm (A.2). Drugs used to treat fasciolosis included TCBZ, closantel and clorsulon. Twenty individual samples were collected per farm from groups of cattle which had not received any fluke treatment for at least 13 weeks, in line with previous studies investigating herd-level diagnostics for *F. hepatica* in sheep and cattle.^24 33^ To reduce the likelihood of resampling the same animals, only freshly voided samples were collected when walking in a zig-zag pattern across the pasture where animals were currently grazing.

Individual fluke egg counts were based on 10 g faecal samples. Composite fluke egg counts were based on 10 individual 5 g samples combined into a 50 g composite sample as previously described for sheep.^24^ Two composite samples were carried out per farm for farms A.1 to A.7, giving 14 composite egg counts in total.

To enable further validation and model parameterisation, including investigation of the effect of quantity of faeces used on test performance, additional individual egg counts, based on 1 g faecal samples (n=500), were taken from 22 fluke-positive UK dairy farms (B.1–B.22), with 20–33 samples per farm collected as part of a previous investigation.^33^

The relationship between composite and individual counts was investigated by fitting negative binomial distributions (NBDs)^24^ to observed individual egg count data for each farm (A.1–A.7 and B.1–B.22, n=29) using maximum likelihood.^36^ The resulting mean (*µ*) and *k* values describing the arithmetic mean and overdispersion of the fitted distributions, respectively, were used to generate NBDs for each farm using the Pop Tools Add-in for Microsoft Excel. Fitted NBDs were checked against the original count data for goodness-of-fit using chi-squared tests (table 1).

**Table 1:** Parameters of fitted negative binomial distributions (NBDs) for farms A.1–A.7 and B.1–B.22

Farm ID	*k*	*µ*	*Χ*^2^ P value
A.1	1.048	14.750	0.194
A.2	0.914	13.850	0.297
A.3	3.905	10.150	0.276
A.4	0.594	0.400	0.878
A.5	2.053	48.667	0.262
A.6	2.152	47.550	0.253
A.7	0.488	2.500	0.878
B.1	0.237	2.077	0.998
B.2	100	0.083	0.925
B.3	0.555	2.320	0.827
B.4	0.825	2.071	0.319
B.5	3.121	4.720	0.759
B.6	1.352	1.640	0.852
B.7	0.252	0.360	0.967
B.8	0.891	2.920	0.834
B.9	0.193	1.625	0.992
B.10	0.811	0.550	0.614
B.11	100	0.400	0.467
B.12	0.240	1.250	0.350
B.13	8.364	1.450	0.667
B.14	0.307	0.950	0.754
B.15	0.675	0.800	0.687
B.16	0.867	2.350	0.676
B.17	100	0.030	1.000
B.18	100	0.150	0.349
B.19	0.020	0.250	1
B.20	0.044	0.100	1
B.21	100	0.100	0.574
B.22	1.097	2.000	0.707

*k* captures the degree of overdispersion and *µ* the mean egg count for each fitted NBD. *X*^2^ P values are the result of comparison between original fluke egg counts and NBD-predicted fluke egg counts.

NBDs fitted to individual egg counts were stochastically resampled, 10,000 iterations per farm. With each iteration, 10 randomly generated NBD-predicted individual count values were summed to give a NBD-predicted composite count. The resulting mean NBD-predicted composite count and SD for each farm was then used to generate 95 per cent CIs against which the original observed composite counts and summed individual count values could be compared. This was achieved as follows:

i. To allow comparison with observed 5×10 g composite count data, NBD-predicted composite counts were converted to number of eggs per 50 g sample: predicted composite counts of 10×10 g individual faecal samples (A.1–A.7) were divided by 2, while predicted composite counts of 10×1 g faecal samples (B.1–B.22) were multiplied by 5.

ii. A coefficient of variation (*CV*), for each farm (n=29; farms A.1–A.7 and B.1–B.22) was calculated from the mean ($x_{1}$) and SD (σ) of the NBD-predicted composite counts:

$$CV=\sigma ÷x_{1}$$

iii. *CV* was modelled as a response variable (*Y*) using multivariable linear regression with data from all 29 farms. Mean NBD-predicted composite count ($x_{1}$) and the quantity of faeces used ($x_{2}$; 10 and 1 g for counts from farms A and B, respectively) were included as explanatory variables. All variables were log-transformed to ensure linear fit (table 2).

iv. The resulting intercept (*α*) and coefficient value ($\beta_{n}$) for each explanatory variable ($x_{n}$) (table 2) was then used to define *CV* and calculate the SD (*σ*) at any given NBD-predicted composite count ($x_{1}$) as follows:

$$\sigma =x_{1}\times e^{\;\alpha +\beta_{1}\left(\log\left(x_{1}+1\right)\right)+\beta_{2}\left(log\left(x_{2}\right)\right)}$$

v. This function was used to generate 95 per cent CIs (±1.96σ) for NBD-predicted composite egg counts $\left(x_{1}\right)$ ranging from 0 to 250 eggs per 50 g composite sample. Observed composite counts for farms A.1–A.7 were compared with this NBD-predicted range when quantity of faeces ($x_{2}$) taken was 5 g. Additionally, the sum of 10 individual counts (per 50 g sample) for all farms from A and B were also compared with these final model parameters when quantity of faeces ($x_{2}$) for NBD-predicted counts was 10 and 1 g, respectively.

**Table 2:** Summary of multivariable linear regression analysis using egg count data from all 29 farms (A and B) in part 1

Variable	Transformation	Intercept (*α*)	Coefficient (*β_n_*)	P value
Response variable (*Y*):				
Coefficient of variation	log(*Y*)	0.779	–	0.0003
Explanatory variables (*x_n_*):				
NBD-predicted FEC (50 g)	log(*x*_1_+ 1)	–	−0.342	1.51×10^−7^
Quantity of faeces (g)	log(*x*_2_)	–	−0.261	0.0004

$log\left(Y\right)=\alpha +\beta_{1}\left(log\left(x_{1}+1\right)\right)+\beta_{2}\left(log\left(x_{2}\right)\right)$ with fitted intercept (*α*), coefficients (*β*) and P values.

FEC, faecal egg counts; NBD, negative binomial distribution.

The model-predicted lower 95 per cent CI calculated for 105 g composite samples (where *x*_2_=5 g) was then used to identify the mean NBD-predicted composite egg count (*x*_1_) at which 95 per cent sensitivity was achieved.

#### Development of a cattle-specific composite sample method

Cattle faeces are normally of a much higher water content, and lower fluke egg concentration compared with sheep faeces. Here, the authors aimed to develop a practical, cattle-specific composite sample field test to provide a qualitative diagnosis representative of herd-level infection status.

In order to collect reliable parameter estimates for distributions of eggs in cattle herds, and investigate any seasonal effects on these, five beef and two dairy herds were sampled. Forty individual animals were sampled from five Scottish beef herds, on three occasions (early spring, late spring/early summer and late summer/autumn); samples were collected after animals had been observed to void faeces. Fifty cows from each of two Cheshire dairy herds were also sampled on one occasion. Individual fluke egg counts were based on 10 g faecal samples. To ensure sampling reflected genuine presence of patent infection, only groups of cattle which had not received any fluke treatment for at least 13 weeks were sampled on each occasion. Due to the need for any test developed to be practical in a clinical setting, composite fluke egg counts and analyses were based on 10 individual 10 g samples (100 g composite sample) from which a 10 g subsample was taken for sedimentation following thorough mixing. This quantity was chosen after preliminary investigations showed further increasing the amount of faecal material used for sedimentation increased processing time and difficulty without greatly improving egg detection (Skuce and others, *unpublished data*).

To develop an appropriate cattle-specific composite analysis method, NBDs were fitted to observed individual faecal egg count sedimentation data using maximum likelihood^36^ and the goodness-of-fit was tested in a chi-squared test. Initially, the influence of the number of individual samples to be included in the composite which would provide a likelihood of 95 per cent of finding greater than or equal to two eggs was assessed. Fitted distributions were Monte Carlo resampled (10,000 iterations) and, for NBD-predicted composites containing 5–50 samples, the proportion of resamplings yielding greater than or equal to two eggs logged. Following consultation with the project team, which comprised industry experts representing the four devolved red meat levy boards and the dairy industry, as well as academics, it was felt that farmers would be unlikely to collect more than 10 individual samples for a ‘simple test’. As a result, it was decided to simulate test sensitivity for a composite sample containing 10 individual samples.

Ten thousand subsets of 10 NBD-predicted sample counts were randomly generated from the fitted distributions and the proportion of resamplings yielding greater than or equal to  two eggs logged. The mean sensitivity and its 95 per cent CIs were bootstrapped (10,000 iterations) from the NBD-predicted values generated for each of the samplings. In some of these, not enough eggs were returned to reliably fit a NBD, yet their inclusion is pertinent to the estimation of overall egg recovery and test sensitivity. In such cases, egg recovery and ‘test sensitivity’ were, therefore, estimated directly from random resamplings of the original observed count data. It was acknowledged that, if samples are to be picked up randomly from the floor by farmers not paying due attention to the freshness of the faeces, certain cows may end up being included in the composite more than once. Therefore, the above random NBD sampling procedure was repeated for eight individual animals with two of these (randomly chosen) being included twice (to give a composite of 10 samples, ‘8+2 sampling’). Last, to investigate whether including two more samples would significantly improve test sensitivity, this NBD sampling procedure was repeated for 12 individual samples.

## Results

### Comparing composite with individual faecal sample analysis

Positive fluke egg counts were found on all farms sampled, with prevalence determined by individual sample egg counts on farms A.1–A.7 ranging from 25 per cent to 100 per cent. Observed composite egg counts for these farms ranged from 0 to 341 eggs per 50 g composite sample (table 3). NBDs fitted to egg count distributions from each farm (A.1–A.7 and B.1–B.22) produced counts representative of the original data, with no significant differences observed (*X*^2^ P≥0.194); fitted mean and *k* values are given in table 1.

**Table 3:** Summary of composite egg counts for farms A.1–A.7

ID	% of egg positive samples*	Composite egg count (10×5 g)
A.1	95	96
		82
A.2	89	61
		46
A.3	100	68
		57
A.4	25	6
		1
A.5	100	167
		341
A.6	100	201
		269
A.7	53	0
		12

Two composite counts are shown for each farm (n=14).

*Based on individual counts.

Following stochastic resampling of fitted NBDs and generation of *CV* values for each farm, multivariable linear regression analysis showed that both (logged) NBD-predicted composite counts ($x_{1}$) and quantity of faeces ($x_{2}$) were significantly negatively correlated with (logged) *CV* (P<0.001 for both variables), yielding an adjusted R^2^ of 0.718 for overall model fit (table 2).

**Figure 1:**
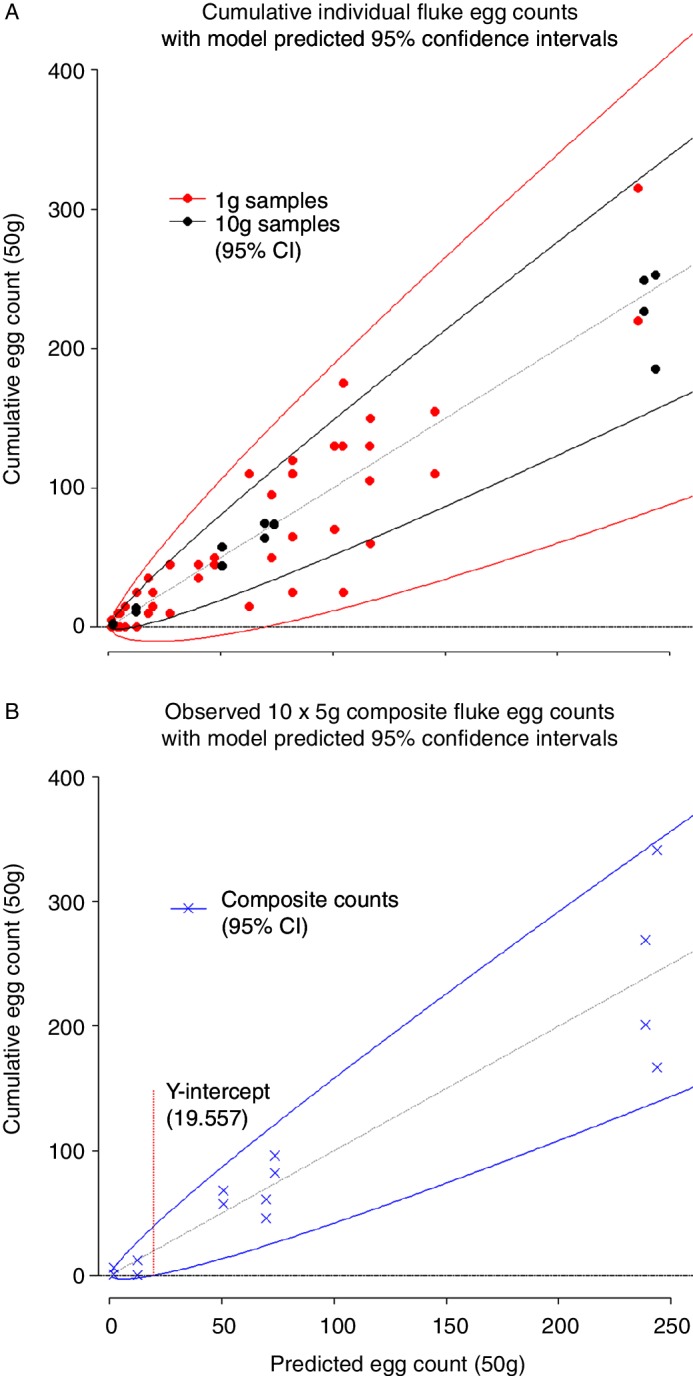
Comparison of model generated 95% CIs from phase I to (a) sum of observed individual egg counts (per 50 g) based on individual counts for 1 and 10 g faecal samples and (b) observed composite egg counts for 10×5 g composite samples.

All values for the sum of observed individual egg counts (per 50 g) based on both 10 and 1 g faecal samples fell within the CIs generated by the model when quantity of faeces ($x_{2}$) was taken to be 10 and 1 g, respectively (figure 1A). Similarly, all observed composite counts from farms A.1 to A.7 fell within CIs generated from individual farm egg count data in the final parameterised model when $x_{2}$ was taken to be 5 g (figure 1B).

Where $x_{2}$ was taken to be 5 g, the lower CI was found to equal zero when mean NBD-predicted composite count was 19.56, indicating a 95 per cent sensitivity at ≥20 eggs per 50 g faeces (≥0.4 eggs/g).

### Development of a cattle-specific composite sample method

The differences observed in egg recovery rates, and the distribution of eggs within the population, varied considerably between farms and between seasons on one farm. On sampled farms where *F. hepatica* was shown to be present through postmortem investigation or previous cattle testing, samplings yielded insufficient egg count numbers to allow NBD-fitting on three occasions, while for one sampling, no eggs were detected at all on one farm (table 4). No clear seasonal pattern could be detected. NBDs provided a good fit for all sampling occasions where a distribution could be fitted (*X*^2^ P≥0.352); fitted mean and *k* values are given in table 4. The Monte Carlo analysis of the number of samples to be included in the composite to achieve 95 per cent sensitivity, therefore, varied dramatically even between two samplings on one farm. For example, on farm I (table 4), for both the March and October samplings, bootstrap analysis of fitted NBDs showed that including 5 samples would be enough to achieve 95 per cent sensitivity, whereas for the June sampling, as many as 35 samples were predicted to be required. The sensitivity of the expert panel designed 10-sample test was assessed in all further analyses. The predicted number of eggs to be found in the composite test, and the likelihood of finding greater than or equal to  two 2 eggs, is given in table 4. Across all samplings, the bootstrapped mean likelihood of finding greater than or equal to  two 2 eggs in a 10-sample composite was 0.69 (95 per cent CI 0.50 to 0.85) (table 5). Neither the inclusion of two randomly chosen individual samples twice (8+2, 10 in total) nor the inclusion of two extra samples (10+2, 12 in total) significantly influenced this approximation of test sensitivity (table 5).

**Table 4:** NBDs fitted to egg count data (≥40 cattle), the predicted numbers of eggs recovered and the probability of finding ≥2 eggs in a 10-sample composite in phase II

Sampling	Parameters	Herd H	Herd M	Herd C	Herd I	Herd N	Herd D	Herd T
March/April	*k*	1.00	0.37	0.41	0.33	NEE	NS	NS
	*µ*	7.06	0.78	0.63	4.08			
	Mean rec	71 (30– 123)	7 (0–21)	6 (1–15)	42 (9–97)	0 (0–2)		
	Probability	1	0.96	0.92	0.99	0.09		
May/ June / July	*k*	0.32	NEE	0.27	0.16	NED	NS	NS
	*µ*	1.68		0.48	0.10			
	Mean rec		1 (0–3)	5(0–14)	1 (0–4)	0 (0–0)		
	Probability	0.99	0.24	0.81	0.25	0		
August	*k*	NS	NS	NS	NS	NS	0.35	0.29
	*µ*						0.46	0.24
	Mean rec						5 (0–13)	2 (0–7)
	Probability						0.89	0.60
September/ October/ November	*k*	0.68	NEE	0.54	0.40	0.30	NS	NS
	*µ*	1.00		0.73	2.48	0.39		
	Mean rec	10 (3–21)	1 (0–3)	7 (1–17)	24 (5–56)	4 (0–11)		
	Probability	0.99	0.25	0.95	0.99	0.77		

*k* and *µ* give the degree of overdispersion and the mean of the fitted NBDs, respectively; ‘mean rec’=simulated mean recovery (95% CI) numbers of eggs in a 10-sample composite; ‘probability’=likelihood of finding ≥2 eggs in the composite sediment.

NBD,  negative binomial distribution; NEE, not enough eggs detected to reliably fit an NBD; NED, no eggs detected; NS, not sampled.

**Table 5:** Bootstrapped estimated egg recovery and likelihood of finding ≥2 eggs across all samplings in phase II

Number of samples	8+2	10	12
Mean rec	12 (4 to 22)	13 (5 to 23)	15 (7 to 27)
Probability	0.68 (0.51 to 0.83)	0.69 (0.50 to 0.85)	0.72 (0.57 to 0.88)

'8+2’=8 random samples and two of these (randomly chosen) contribute to the composite twice; ‘mean rec’=simulated mean recovery (95% CI) numbers of eggs in a 10-sample composite; ‘probability’=likelihood of finding ≥2 eggs in the composite sediment (95% CI), eg,  the estimated overall sensitivity of the test.

## Discussion

This study investigated two distinct aspects of FEC sedimentation testing of cattle faeces. First, the authors determined whether a composite sample can be as sensitive as individual samples. Second, the authors assessed whether a composite test can be a sensitive farm-level diagnostic tool confirming the qualitative presence of *F. hepatica* on cattle farms.

In the first part of this study, through the use of statistical modelling, the authors have demonstrated that fluke egg counts based on composite samples correspond closely, and in a linear fashion, to those expected when performing sedimentations on separate individual samples. With respect to its usefulness as a qualitative test, composite sample analysis was capable of detecting eggs with 95 per cent certainty when individual sedimentation counts were less than 1 egg/g of faeces. The validity of this model-based approach is supported by the observation that all composite counts (n=14) from farms A.1 to A.7 fell within the NBD-generated CIs, as did the sum of observed individual egg counts (per 50 g) for all 29 farms, suggesting the final parameterised model was representative of actual observations.

With respect to the second part of this study, the results indicate that in cattle the analysis of a 10 g subsample taken from a 10×10 g composite sample has an estimated sensitivity of 0.69, with a lower 95 per cent confidence limit of 0.50. This analysis also showed that accidentally including the dung of two animals twice when randomly picking up samples deposited at pasture was predicted to have very little effect on test sensitivity, indicating the robustness of the proposed method.

When considering the test sensitivity of composite sample methods, it is important to note that for diagnosis of fluke infection in individuals, egg count methods are known to have a relatively low sensitivity.^20 23^ This may be for a variety of reasons including (but not limited to) prepatent infection and low fluke egg concentrations within submitted faecal samples, with the latter point of particular note in cattle compared with sheep. Furthermore, the seasonal nature of *F. hepatica* transmission in temperate climates and the inability of faecal egg counts to detect prepatent infection results in variation of test sensitivity at different times of year.^20 34^ Lower sensitivity values are reported during periods of peak pasture infectivity (ie, autumn) due to the relative increase in juvenile infections occurring at this time.

An overall diagnostic sensitivity of 0.69 for herd-level diagnosis by composite sample analysis is comparable to previously published sensitivity values for fluke egg counts when diagnosing individual cattle.^20 23 34^ Despite this relatively low sensitivity, fluke egg counts are still an important diagnostic tool for reasons previously discussed relating to their high specificity, provided treatment history is known, and the low cost and ease of processing. Furthermore, in the case of the composite sample egg count method, there is no single sample test alternative available for use in the herd-level diagnosis of beef and non-lactating dairy cattle. As part of the current investigation, test results from a herd-level composite diagnostic cELISA indicate that this method was less sensitive than the equivalent composite egg count using the analysis method described (Skuce and others, *unpublished data*), although it must be noted, this initial analysis was performed with the original Bio X cELISA kit.

Consequently, a diagnostic field test based on a composite sample composed of 10×10 g individual samples represents a simple practical test for cattle herds, with the caveat that a negative test should always be followed up with a second test. Ideally, such retesting should be carried out in autumn or early spring, with the expectation that any infections acquired over the previous grazing season will have become patent by this time. Furthermore, due to the dependence of this method on the presence of patent infection, this test can and should only be relied on either in untreated cattle, or animals which have been treated with a flukicide and exposed to re-infection more than 13 weeks previously.

It should be stressed that for the second part of this study, herd sampling occurred in a dry year with very low overall fluke abundance. Concurrent pasture surveys aiming to identify (infected) *Galba truncatula* intermediate host snail populations largely failed to identify any snails that year (O’Hare and others, *unpublished data*). The calculated test sensitivity given here is, therefore, likely to reflect the worst-case scenario, since fluke infection and egg counts under such conditions are likely to have been lower than what would be typically expected. As such, this test represents a simple yet effective diagnostic tool for identifying *F. hepatica* infection in cattle herds. Furthermore, while this study focused on the diagnosis of *F. hepatica*, the sedimentation method described here may also be useful in the detection of eggs of other trematodes, including *F. gigantica* and paramphistomes (rumen fluke), such as *Calicophoron daubneyi*,^22^  reports of which have become increasingly common in the UK and Ireland in recent years.

Designing a ‘workable’ composite test that is likely to be used by farmers and sufficiently sensitive to identify *F. hepatica* on a farm with high certainty is far from straightforward. In line with what was found in sheep,^24^ fluke infections in cattle appeared more heavily overdispersed than gastrointestinal nematode infections.^37^ Therefore, the level of egg recovery, and the probability of finding eggs at all, depend very strongly on which animals are included in the sample. Further work could investigate whether using larger amounts of faeces from each animal (12–15 g) will result in higher test sensitivity. In addition to sampling practicalities on farm, such as potential reluctance of farmers to collect more than 10 samples, sample handling practicalities in the laboratory also need to be considered, whereby the advantages of increased sample volume for individual or composite samples may be offset by the negative impact of the time needed to create and test the composite sample.

It is to be expected that due to factors such as pasture quality and grazing management, the level of egg excretion varies widely between beef farms in the same locality (eg, postcode area) sampled at the same time of year. Within individual farms, egg excretion levels and the level of overdispersion showed strong seasonal variation. On one occasion, on a farm where fluke was present (identified previously through postmortem investigation or cattle testing and confirmed in other samplings), even sampling and processing of more than 40 individual animal samples did not return a single egg. Although a clear seasonal pattern was not present, the results suggest test sensitivity may be higher during early spring and in autumn when compared with other times of year as has been demonstrated previously in the UK and elsewhere.^20 34^ It is important to note, however, that previous investigation of acute (juvenile) versus chronic (adult) stage infection in cattle from the UK and Republic of Ireland over the autumn months has shown adult infections to be present in the majority of instances,^38^ indicating faecal egg counts are still a useful and reliable diagnostic tool in the majority of cases.

Despite the variability in egg counts between samplings, probability-based analyses would be strongly placed to aid the design of a highly sensitive test. However, the resulting presence-absence test would likely have contained many more individual samples than would be practical, negating its uptake. The involvement of industry representatives in discussions around test requirements and practical considerations was, therefore, highly beneficial at this stage of test development, since it led to a more pragmatic approach being adopted with the development of a composite sample limited to a maximum of 10 fresh individual samples.

In summary, through a combination of experimental and statistical modelling techniques the authors have provided evidence of the validity of composite faecal egg samples in the qualitative diagnosis of *F. hepatica* in cattle via a simple sedimentation method. The authors have demonstrated that composite sample analysis is both equivalent to individual sample analyses, and is representative of herd-level infection status with a diagnostic sensitivity of 0.69 (95% CI 0.5 to 0.85). The authors conclude that composite faecal egg sedimentation in cattle provides a simple but effective diagnostic test to aid the control of fluke infections, particularly since egg sedimentation can be performed easily in most veterinary practices.
